# Multilevel modelling of factors associated with caesarean section in Ethiopia: community based cross sectional study

**DOI:** 10.1186/s13104-019-4705-2

**Published:** 2019-11-06

**Authors:** Abebaw Gedef Azene, Abiba Mihret Aragaw, Mihretie Gedefaw Birlie

**Affiliations:** 1 Department of Epidemiology and Biostatistics, Bahir Dar University, Bahir Dar, Ethiopia; 2 Department of Statistics, Debre Markos University, Debre Markos, Ethiopia; 3 Department of Nursing, Debre Markos University, Debre Markos, Ethiopia

**Keywords:** Caesarean section, Multilevel modelling, Prevalence, Ethiopia

## Abstract

**Objective:**

The aim of this study was to identify socio-demographic and health related factors associated with caesarean section in Ethiopia.

**Results:**

A total of 256 mothers undergoing to CS among 7193 delivery. Average maternal age of a participant was 29.26 years and 80% of mothers having two and more children. A woman delivered in private institution was 30% (AOR = 1.29; 95% CI 1.25, 1.32) more likely undergoing CS as compared to home delivery. Factors associated with CS were higher education level (AOR = 1.09, 95% CI 1.07, 1.12), preceding birth interval (AOR = 1.01; 95% CI 1.00. 1.03), multiple pregnancy (AOR = 1.11; 95% CI 1.08, 1.15), multiple parity (AOR = 0.98; 95% CI 0.97, 0.99), large size the child (AOR = 1.01; 95% CI 1.001, 1.02), richest households (AOR = 0.98; 95% CI 0.97, 0.99), rural residence (AOR = 0.98 95% CI 0.96, 0.99) and Addis Ababa (AOR = 1.06; 95% CI 1.04,1.09). As a conclusion and recommendation, the prevalence of CS higher in private institutions and Addis Ababa, so professionals should apply CS alone medical indication.

## Introduction

Caesarean section is an operative technique to deliver a fetus from mothers through abdominal and uterine surgery [1, 2]. Caesarean section has more preferring by mothers in the world [3, 4]. WHO reported that the prevalence of CS were 18.6% worldwide, 7.3% in Africa and small in sub-Shara Africa [5, 6]. In Nigeria, the prevalence of CS was 2.1% [7]. The prevalence of CS varies from 2% up to 64% in Ethiopia [8, 9]. The trend is increasing in Ethiopia [3, 10].

Obstetric care services is improved in the country [11, 12]. Due to this, CS have been performed either by the mothers’ preference or the professional business based recommendation [13–15]. However, Unnecessary CS increases risks on both maternal outcomes and babies [16, 17]. The risks were postpartum morbidity, reduced morbidity, infection, respiratory problem on chilled, less breastfeeding and birth trauma despite of the fact that the prevalence of CS is increasing [18, 19].

Due to the fact that maternal age during delivery, place of delivery, residence, maternal education, ANC, parity, size of the child and multiple pregnancy were factors associated with CS [7, 20].

Studies have been focused on medical indicators of CS. Besides medical indicators, sociodemographic, health factors having a contribution to increase the prevalence of CS in Ethiopia. Therefore; this study intended to assess associated socio-demographic, health factors of CS. This research will be vital to provide some input for health institution.

## Main text

### Methods

#### Study design and setting

Community based cross-sectional study was conducted using 2016 EDHS data collected from January 18 to June 27, 2016. 7,193 women were included for the study. The dataset has a hierarchical structure as a woman nested within geographic regions and residence. The hierarchy for this study follows individuals as level-1, regions and residence as level-2.

#### Inclusion and exclusion criteria

All women (age 15–49) who give birth within five years prior to the study were considered during the 2016 EDHS survey was included.

#### Dependent variable

The outcome variable was a mode of delivery classified as either vaginal delivery (= 0) or CS delivery (= 1).

#### Independent variable

Explanatory variables were classified level-1 and level-2 factors. Level-1 factors were a religion, maternal education, household wealth index, place of delivery, parity, birth order, preceding birth interval, the history of abortion, ANC, anaemic status, multiple pregnancy, drug takes for intestinal disease, sex of the child and size of the child. Level-2 factors were region and residence.

#### Statistical analysis and model

Bivariable analysis was applied to examine the association between each variable and CS. Multilevel logistic regression model was used to comprehend community variation (level-2) and identifying socio-demographic health (level-1) factors associated with CS.

## Results

A total of 7193 mothers were eligible. Out of those mothers, 256 (1.9% weighted by region) were undergoing CS. Average maternal age was 29.26 years, 80% of mothers having more than one children, 8.85% mothers had a history of abortion. Of mothers had a history of abortion, 4.87% were undergoing to CS.

Among mothers undergoing to CS, 54.29% were orthodox, 33.20% were primary school educated, 12.11% had a history of abortion, 9.37% had taken the drug for intestinal disease during pregnancy and 93.75% had multiple pregnancy (see Tables 2 and 3 in Appendix). Regarding level-2 factors, 21.9% were residing in Addis Abeba. 12.7% residing from urban areas (see Table 1).

Among 6937 vaginal delivered mothers, 4317 (62.23%), 2411 (34.76%) and 6331 (91.26%) weren’t educated, from poorest household and hadn’t history of abortion, respectively.

Bivariate analysis of factors associated with CS were conducted. See Table 2 in Appendix and Table 1.

**Table 1:** Bivariate analysis of association between community level factors with CS, EDHS 2016

Covariates	CS n (%)	All vaginal births n (%)	P-value
Region			< 0.0001
Tigray	21 (2.7)	751 (97.3)	
Afar	6 (0.9)	641 (99.1)	
Amhara	16 (2.1)	748 (97.9)	
Oromia	14 (1.4)	1017 (98.6)	
Somali	5 (0.6)	801 (99.4)	
Benshangul	6 (1)	570 (99.0)	
SNNP	23 (2.6)	870 (97.4)	
Gambella	6 (1.1)	528 (98.9)	
Harari	46 (11.2)	365 (88.8)	
Addis Abeba	82 (21.9)	293 (78.1)	
Dire Dewa	31 (8.1)	353 (91.9)	
Residence			0.000
Urban	192 (75)	1320 (19.03)	
Rural	64 (25)	5617 (80.97)	

### Multivarable multilevel logistic regression analysis of CS

Measure of association and random intercepts for CS are presented Table 3 in Appendix. The results of the empty model (Model_1) indicated that there was a significant variation of CS between level-2 factors (random intercept variance = 1.064, P-value = 0.000).

Similarly, the ICC in the empty model implied that 24.44% of the total variance in mode of delivery was credited to differences between level-2 factors.

In Model_2 only level-1 variables were added. With this, maternal education, multiple pregnancy, preceding birth interval, place of delivery, size of the child, parity and household wealth index were significantly associated with CS. ICC in Model_2 indicated that, 23.33% of the variation of CS were accountable to differences across levels-2 factors. As shown by a PCV, 5.9% of the variance in CS across the level-2 was explained by level-1 characteristics.

Model_3, only level-2 factors were added. The result revealed that a woman residing in a rural community was significantly associated with CS. In addition to this; a woman from Somali, Gammbella, Harreri, Addis Abeba and Dire Dewa regions were significantly associated with CS. The ICC showed that differences between level-2 factors account 23.98% of the variation of CS. In addition, PCV indicated that 3.7% of variation of CS explained by level-2 characteristics.

Model_4, the final model included both level-1 and level-2 factors simultaneously which have a P-value less than 0.1 from bivariable analysis. The estimated ICC, 23.35% of the variability in CS were accountable to differences between level-2 factors. PCV indicated that, 3.5% of variation of CS across level-2 explained by both level-1 and level-2 factors. After adjusting the other level-1 and level-2 factors; maternal education, preceding birth interval, place of delivery, multiple pregnancy, size of child, parity and household wealth index in level-1 and also residence and region (Gambella, Hareri, Addis Abeba and Dire Dewa) in level-2 were significantly associated with CS.

The odds of a mother having higher education, undergoing to CS were 9% (AOR = 1.09, 95% CI 1.07, 1.12) more likely compared to mothers who hadn’t educated. The odds of experiencing CS were 1.01 (AOR = 1.01; 95% CI 1.00, 1.03) times more likely for one month increases.

Regarding a woman having multiple pregnancy was 11% (AOR = 1.11; 95% CI 1.08, 1.15) more likely to give birth by CS. Likewise, a woman in public, private and NGO health institution was 1.04 (AOR = 1.04; 95% CI 1.03, 1.05), 1.29 (AOR = 1.29; 95% CI 1.25, 1.32), 1.07 (AOR = 1.07; 95% CI 1.02, 1.12),1.09 (AOR = 1.09; 95% CI 1.05, 1.130) times more likely undergoing to CS as compared to home delivery, respectively. A woman having large child size more likely deliver by CS than medium (AOR = 1.01; 95% CI 1.001, 1.02). A woman having between two and five children was 2% (AOR = 0.98; 95% CI 0.97, 0.99) less likely undergoing to CS as compared to having a primary child.

The odds of a woman from the richest households were 2% (AOR = 0.98; 95% CI 0.97, 0.99) less likely undergoing to CS as compared to a woman from the poorest households. Similarly a woman in rural residence was 2% (AOR = 0.98 95% CI 0.96, 0.99) less likely to give birth by CS as compared to urban counterparts. Looking Region, a woman residing from Harerri had 2% (AOR = 0.98; 95% CI 0.96,0.99) less likely undergoing to CS compared with Tigray. The odds of a woman from Gambella, Addis Abeba and Diere Dewa having more likely undergoing to CS than a woman from Tigray (AOR = 1.02; 95% CI 1.01,1.04), (AOR = 1.06; 95% CI 1.04,1.09) and AOR = 1.08; 95% CI 1.06,1.11) respectively. See Table 3 in Appendix.

## Discussion

Community based cross-sectional study was conducted using 2016 EDHS data collected from January 18 to June 27, 2016. The aim of the study was to identify associated socio-demographic, health factors of CS.

The findings showed that a woman’s having higher educated were more likely undergoing to CS. This could be due to a reason that educated woman search a means to minimize labour and more worry their health. Similar findings were observed a study conducted in Dessie town and Iran [10, 20].

CS was significantly associated with proceeding birth interval. A woman having more proceeding interval; more likely undergoing to CS. This finding is also supported by findings from Eastern Ethiopia and Developing countries [9, 21]. This could be due to a reason relation between the preceding birth interval and uterus.

The other factors associated with CS were multiple pregnancies. A mother faced multiple pregnancy was more likely to deliver by CS compared to single pregnancy. This result agreed with a study conducted in Nigeria and Ethiopia [7, 9]. The possible explanation could be mothers having multiple pregnancies were more sought health institution related with complication of labour.

Place of delivery was significantly associated with CS. A mother delivered in Private, NGO and governmental hospital was more likely undergoing to CS than home delivered woman. Possible explanation could be related with access of CS in health institutions and due to business oriented professional recommendation. This finding also supported a finding from Harerri and Dessie town [9, 10]. The finding revealed that the size of the child associated with CS. A woman having a large size child more likely delivered by CS. This finding supported a finding in Nigeria and Bahir Dar [7, 22]. The possible reason could be due to complication of labour and related to the interval of the uterus.

Mothers having two up to five children were less likely undergoing to CS as compared to primary birth. This finding supported a findings conducted in Egypt and Southwest Ethiopia [23, 24]. A possible reason could be related with experience of a uterus and labour of a woman.

Surprisingly, a study revealed that ANC hadn’t significant association with CS. This finding consistent with a study conducted in Sodo [25]. The reasons could be seek skilled person if they start ANC earlier.

The findings verified that a woman from the richest household had smaller odds undergoing to CS compared to a woman from poorest household. This finding is supported a study conducted at Addis Abeba and Bangladesh [11, 14].This could be due to a reason free service of CS in governmental health institution. In addition to this, mothers from richest household having more access of information from media.

In the study, mothers from rural residence had less likely to deliver by CS than mothers from urban. This finding was agreed a findings conducted in Bangladesh and Nigeria [7, 14]. This could be due to the fact that mothers from rural residence having less chance to access hospitals. In addition to this, most women from a rural residence were poor, both economically and information. But, this finding inconsistent a findings conducted at the Felege Hiwot referral hospital [22, 26].

Similarly, a mother residing from Harerri region were less likely undergoing to CS. However, mothers from Gambella, Addis Abeba, and Diere Dewa were more likely delivered by CS as compared mothers from Tigray region. This findings consistent with the studies conducted in Sodo [25]. This observation may be related to access of health institutions in the region.

## Conclusions

This Study found that 256 mothers out of 7193 were undergoing CS. Most of mothers undergoing to CS were delivered at government health institutions. Factors associated with CS were maternal education, preceding birth interval, multiple pregnancy, place of delivery, parity, the size of the child and household wealth index at individual characteristic were significantly associated with CS. All community factors (Residence and region) were associated with CS. Therefore, the prevalence of CS was varied based on residence and regions in Ethiopia. As a recommendation, the prevalence of CS high in private institutions and Addis Ababa City, so professionals should apply CS alone medical indication.

## Limitation of the study

The study was used secondary data due to this; a respondent’s may answer usually underestimate in socio- demographic factors and overestimate health related factors since the study was retrospective. Primary data is preferable to access obstetric factors. Therefore, the use of this information for comparison and decision-making should consider the inherent limitation of the study.

## Data Availability

The datasets used and/or analysed during the current study available from the corresponding author and EDHS 2016 on reasonable request.

## Appendix

See Tables 2 and 3.

**Table 2:** Bivariate analysis of associated factors at level-1 demographic health variables with CS N = 17,193

Covariates	CS n (%) mean (sd)	All vaginal birth n (%) mean (sd )	P-value
Religion			0.000
Orthodox and Catholic	139 (54.29)	2279 (32.85)	
Protestant	42 (16.41)	1296 (18.68)	
Muslim	74 (28.90)	3250 (46.85)	
Other	1 (0.39)	112 (1.61)	
Education			0.000
Not educated	42 (16.41)	4317 (62.23)	
Primary	85 (33.20)	1857 (26.77)	
Secondary	56 (21.88)	521 (7.51)	
Higher	73 (28.52)	242 (3.49)	
Birth order	2.31 (1.83)	3.98 (2.56)	0.000
Preceding birth interval	60.51 (34.98)	40.95 (23.55)	0.000
Multiple pregnancy			0.000
Single	240 (93.75)	6842 (98.63)	
Multiple	16 (6.25)	160 (1.37)	
Place of delivery			0.000
Home	0 (0%)	1498 (64.5%)	
Government’s institution	179 (69.9%)	2264 (32.6%)	
Private Institution	70 (27.3%)	125 (1.8%)	
NGO Institution	7 (2.7%)	54 (0.8%)	
ANC			0.000
≤ 3	61 (23.8%)	4512 (65%)	
> 3	195 (76.2%)	2425 (35%)	
Size of child			0.001
Large	104 (40.6%)	2059 (29.7%)	
Medium	94 (36.7%)	2896 (41.7%)	
Small	58 (22.7%)	1992 (28.6%)	
Party			0.000
1	115 (44.9%)	1355 (19.5%)	
[2–5]	112 (43.8%)	2978 (42.9%)	
>5	29 (11.3%)	2604 (37.5%)	
Smoking status			0.719
No	254 (99.22)	6867 (98.99)	
Yes	2 (0.78)	70 (1.01)	
Sex of child			0.328
Male	140 (54.69)	3578 (51.58)	
Female	116 (45.31)	3359 (48.42)	
History of abortion			0.062
No	225 (87.89)	6331 (91.26)	
Yes	31 (12.11)	606 (8.74)	
Drugs take for intestinal disease			0.215
No	232 (90.63)	6478 (93.38)	
Yes	24 (9.37)	459 (6.62)	
Anemia			0.001
Sever	2 (0.78)	117 (1.69)	
Moderate	11 (4.30)	659 (9.50)	
Mild	44 (17.19)	1535 (22.12)	
Not anemic	199 (77.73)	4626 (66.69)	
Wealth index			0.000
Poorest	17 (6.64)	2411 (34.76)	
Poorer	12 (4.69)	1167 (16.82)	
Middle	13 (5.07)	1015 (14.63)	
Richer	14 (5.47)	903 (13.02)	
Richest	200 (78.13)	1441 (20.77)

**Table 3:** Multilevel logistic regression analysis of level-1 and level-2 demographic health factors associated with CS, 2016EDHS

Covariates	Model_1 AOR (95%CI)	Model_2 AOR (95%CI)	Model_3 AOR (95%CI)	Model_4 AOR (95%CI)
Education level (not educated)		1		1
Primary		1.01 (0.99, 1.02)		1.01 (0.99, 1.02)
Secondary		1.02 (1.00, 1.03)		1.02 (1.00, 1.03)
Higher		1.10 (1.07, 1.12)*		1.09 (1.07, 1.12)*
Birth order		1.00 (1.00, 1.00)		1.00 (1.00, 1.00)
Preceding birth interval		1.00 (1.00, 1.00)*		1.00 (1.00. 1.00)*
Child is twin (single)		1		1
Multiple		1.11 (1.08, 1.15)*		1.11 (1.08, 1.15)*
Place of delivery (home)		1		
Government institution		1.04 (1.03, 1.05)*		1.04 (1.03, 1.05)*
Private Institution		1.29 (1.26,1.33)*		1.29 (1.25, 1.32)*
NGO Institution		1.07 (1.03, 1.12)*		1.07 (1.02, 1.12)*
History of abortion (no)				
Yes		1.01 (0.99, 1.02)		1.01 (0.99, 1.02)
ANC (≤ 3)				
>3		1.01 (1.00, 1.02)		1.01 (1.00, 1.02)
Size of child (large)				
Medium		0.99 (0.98, 0.99)*		0.99 (0.98, 1.00)*
Small		0.99 (0.98, 1.01)		0.99 (0.98, 1.01)
Parity (1)				
[2–5]		0.98 (0.97, 0.99)*		0.98 (0.97, 0.99)*
>5		0.98 (0.96, 1.00)		0.98 (0.96, 1.00)
Anemic status (sever)		1		1
Moderate		0.99 (0.96, 1.03)		0.99 (0.96, 1.03)
Mild		1.00 (0.97, 1.03)		1.00 (0.96, 1.03)
Not anemic		1.00 (0.96, 1.03)		0.99 (0.96, 1.03)
Household wealth index (poorest)		1		1
Poorer		0.99 (0.98, 1.00)		0.99 (0.98, 1.00)
Middle		0.99 (0.97, 1.00)		0.99 (0.97, 0.99)*
Richer		0.98 (0.97, 0.99)*		0.98 (0.97, 0.99)*
Richest		1.01 (1.00, 1.03)		1.00 (0.98, 1.02)
Residance (Urban)			1	1
Rural			0.93 (0.91, 0.94)*	0.98 (0.96, 0.99)*
Region (Tigray)			1	1
Afar			0.99 (0.97, 1.01)	1.01 (0.98,1.05)
Amhara			0.99 (0.98, 1.02)	1.00 (0.99,1.02)
Oromia			0.99 (0.97, 1.01)	1.02 (1.00,1.03)
Somali			0.98 (0.96, 0.99)*	1.01 (0.99,1.03)
Benshangul			0.99 (0.97, 1.01)	1.00 (0.98,1.02)
SNNP			1.005 (0.98, 1.02)	1.01 (0.99,1.03)
Gambella			0.98 (0.96, 0.99)*	1.02 (1.01,1.04)*
Hareri			1.07 (1.05, 1.09)*	0.98 (0.96,0.99)*
Addis Abeba			1.14 (1.11, 1.18)*	1.06 (1.04,1.09)*
Dire Dewa			1.03 (1.004, 1.05)*	1.08 (1.06,1.11)*
Communities variance (se)	1.064 (0.602)	1.001 (0.17)	1.038 (0.037)	1.002 (0.17)
ICC	24.44%	23.33%	23.98%	23.35%
PCV	Ref	5.92%	3.7%	3.5%
Model fit				
log likelihood	2169.56	2540.081	2268.38	2559.534
AIC	−4333.119	− 5030.163	− 4510.76	− 5051.068

*Significant at P-value < 0.05

## Abbreviations

ANC: antenatal care
EDHS: ethiopian demographic health survey
DHS: demographic health survey
CS: caesarean section
EA: enumerate Area
sd: standard deviation
